# Evaluating methane inventories by isotopic analysis in the London region

**DOI:** 10.1038/s41598-017-04802-6

**Published:** 2017-07-07

**Authors:** G. Zazzeri, D. Lowry, R. E. Fisher, J. L. France, M. Lanoisellé, C. S. B. Grimmond, E. G. Nisbet

**Affiliations:** 1 Royal Holloway University of London, Egham Hill, Egham, UK TW20 0EX United Kingdom; 2 University of East Anglia, Norwich Research Park, Norwich, Norfolk NR4 7TJ UK; 3 University of Reading, Reading, RG6 6BB UK; 4 Imperial College London, Kensington, London SW7 2AZ UK

## Abstract

A thorough understanding of methane sources is necessary to accomplish methane reduction targets. Urban environments, where a large variety of methane sources coexist, are one of the most complex areas to investigate. Methane sources are characterised by specific δ^13^C-CH_4_ signatures, so high precision stable isotope analysis of atmospheric methane can be used to give a better understanding of urban sources and their partition in a source mix. Diurnal measurements of methane and carbon dioxide mole fraction, and isotopic values at King’s College London, enabled assessment of the isotopic signal of the source mix in central London. Surveys with a mobile measurement system in the London region were also carried out for detection of methane plumes at near ground level, in order to evaluate the spatial allocation of sources suggested by the inventories. The measured isotopic signal in central London (−45.7 ±0.5‰) was more than 2‰ higher than the isotopic value calculated using emission inventories and updated δ^13^C-CH_4_ signatures. Besides, during the mobile surveys, many gas leaks were identified that are not included in the inventories. This suggests that a revision of the source distribution given by the emission inventories is needed.

## Introduction

Accurate quantification of methane emissions both at global and regional scale and full understanding of methane sources are still unresolved issues. The emission estimates produced by bottom-up and top-down approaches can be highly discordant^1^. The evaluation of bottom-up estimates, which are based on statistics multiplied by specific emission factors, through atmospheric observations (“top-down” approach) demands enhanced measurement techniques and a well distributed network of measurement stations^2^. However, while many specific approaches have been developed for the estimate of total methane fluxes (e.g. refs 3–7), there is no established methodology that leads to a comprehensive partitioning of emissions by sources. Tracer methods based on Fourier Transform Infrared (FTIR) absorption spectroscopy^8^, flux chambers^9^ and mobile ground surveys^10^ are all used for the estimation of total emissions from a specific source area (e.g. landfill sites), but they do not discriminate between different methane production processes. Conversely, the stable isotopic analysis provides insight on the methane origin, enabling distinction between biogenic (^13^C depleted), thermogenic and pyrogenic methane sources (^13^C enriched), and their partition in a source mix^11–16^.

Urban environments, where a large variety of methane sources coexist, such as leaks in the natural gas distribution network, landfill sites and sewage works, are undoubtedly the most complex areas to investigate. The Greater London region, which covers ~0.6% of the UK area, produces approximately 3% of the total methane emissions in the UK according to bottom- up inventories^17^.

A preliminary overview of sources can be achieved thanks to the accessibility of official inventory data, which in the UK is provided by the National Atmospheric Emissions Inventory (NAEI), where datasets with estimates of the distribution of methane emissions at high resolution (1 km^2^) are available. Nevertheless, the spatial distribution of sources is fairly coarse and estimates are provided without an error assessment. In particular, emissions from source areas such as landfill sites and sewage works can be widely distributed, and their spatial allocation highly uncertain. An independent evaluation through atmospheric observations is therefore critical.

In this study, the isotopic analysis of the methane source mix in central London, coupled with continuous methane and carbon dioxide mole fraction measurements carried out during diurnal studies at King’s College London (KCL), has permitted further understanding of the methane source mix in the London basin, integrating the analysis of the source proportion previously suggested by the studies of Lowry *et al*.^13^ and Fisher^18^ at Royal Holloway University of London (RHUL).

The spatial distribution of sources in the inventories has been tested for the London borough of Hounslow and the central district of Camden Town, offering an example of how local sources can be identified and, through their isotopic characterisation, carefully distinguished when they emit concurrently in the same area.

## Methods

### Typical δ^13^C-CH_4_ signatures and CH_4_ emission and isotopic maps

Typical δ^13^C signatures have been attributed to the major methane sources in SE England in the study of Zazzeri^19^, through the identification and sampling of methane emission plumes and the calculation of a source isotopic signature by Keeling plot analysis. The revised δ^13^C-CH_4_ values reflect the temporal variability of the sources, being the result of extensive measurement campaigns at many sites and at different times of the year. These δ^13^C-CH_4_ signatures were assigned to the UNECE (United Nations Economic Commission for Europe) source sectors used for categorising methane emissions inventories (Table 1).

**Table 1:** δ^13^C signatures of UK methane sources, based on measured values^13, 18^ and literature review.

UK Methane Sources	Emission ktonne (2013)	UNECE Sector	Typicalδ^13^C-CH4 (‰)
Enteric fermentation (cows)	945	Agriculture	−66*
Waste disposal and landfills	696	Waste treatment and disposal	−58*
Gas transmission and distribution	218	Offshore	−36*
Manure management	139	Agriculture	−58
Wastewater handling	134	Waste treatment and disposal	−53*
Coal mining	64	Offshore	−45*
Combustion (industrial and domestic)	44	Combustion in energy production and transfer	−25
Road transport	2	Road Transport	−20
Biomass burning	1	Agricultural burning and wildfires	−28
UK Total	2243		−58

^*^Indicates values that have been revised by the study of Zazzeri^19^. 2013 NAEI emission inventories are provided without errors. The agriculture sector includes both enteric fermentation and manure; offshore category refers to emissions from fossil fuels (natural gas and coal); waste treatment sector includes both landfill sites and sewage works. The natural gas isotopic value of −36‰ has been attributed to the “offshore” sector, as no coal mines are located within the London area. Errors of measured isotopic values are within ±3‰.

**Figure 1:**
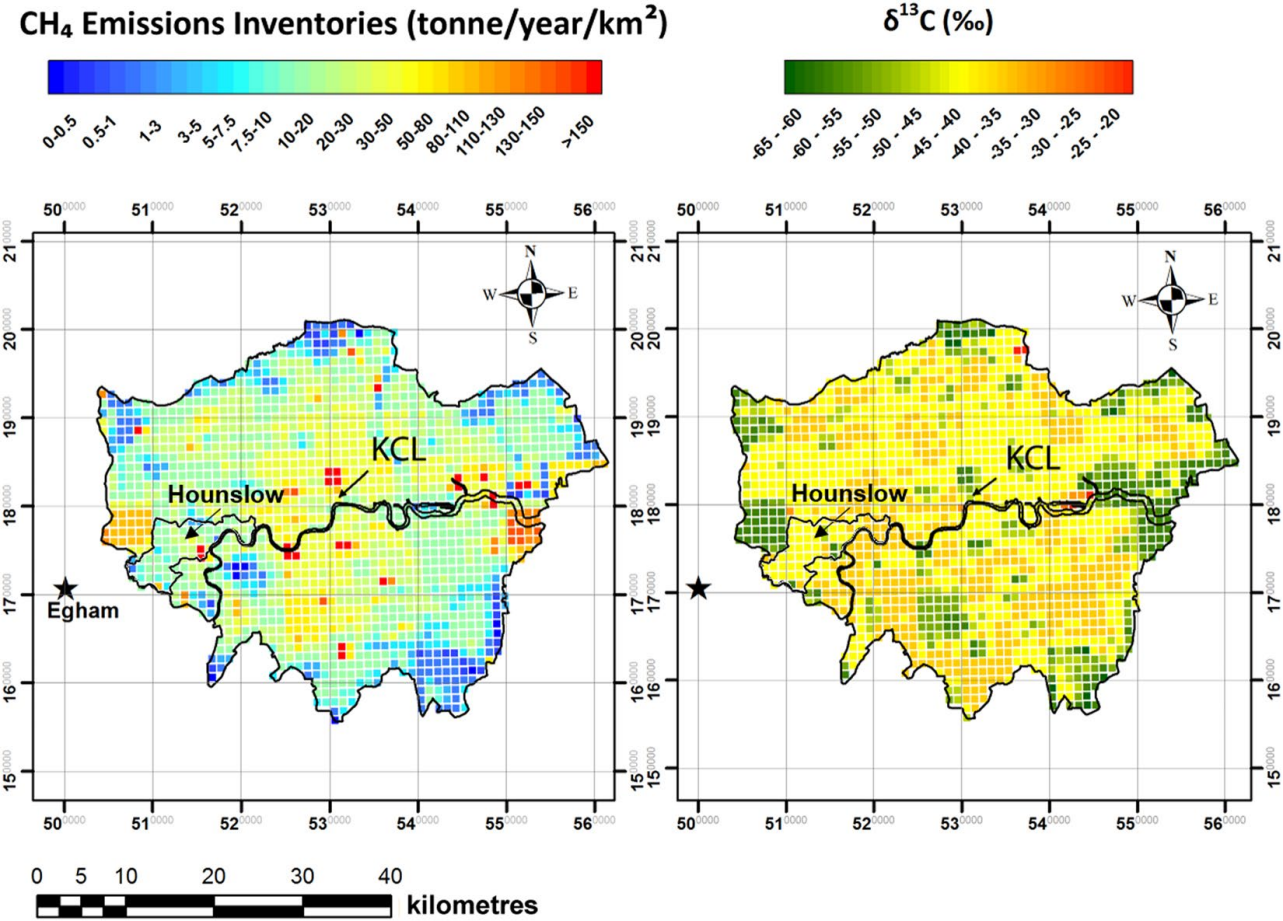
Emission (**a**) and isotopic (**b**) map for London based on 2013 NAEI methane inventories. Emissions are provided in tonne/year/km^2^ (~3.2 · 10^−11^ kg/s/m^2^). The star indicates Egham, where the Royal Holloway University of London is located. King’s College London (KCL) and the borough of Hounslow are indicated. Most of the hotspots are associated with ^13^C-depleted areas. The large area affected by high emissions on the east side of the London region includes biogenic methane sources, such as landfill sites and waste-water treatment plants. The map was generated using ArcMap 10.2.

Methane emissions at 1 × 1 km resolution are available for each UNECE sector (Table 1) in the National Atmospheric Emissions Inventory (NAEI). Using the isotopic values (Table 1), and computing a weighted average of δ^13^C-CH_4_ for each km^2^, emissions data have been converted to isotopic signatures (Fig. 1b) for comparison with the emission map (Fig. 1a). The calculated isotopic signature for each grid square is the expected isotopic signal of the emissions from that grid cell, based on the mix of sources assessed in the emission inventories.

### Diurnal Studies in London

Collection of air samples for detailed diurnal studies was carried out through an automatic sampler (see supplementary material) installed on the rooftop at the Strand Campus of KCL (51°30′43″ N, 0°07′00″ W), when a large overnight build-up in mole fraction was expected (i.e. anticyclonic weather conditions over the London basin). The automatic sampler was connected to an air inlet placed at 7 metres above roof height and 48.6 m above ground level on a triangular tower (Aluma T45-H), and allowed up to 20 Tedlar or Flexfoil bags to be filled. The sampling time was arranged to cover a full 24 hours (~1 sample every 72 min), including the late-afternoon rush hour and the early morning mole fractions build-up (see supplementary material for the time interval and filling time setting).

The Strand Campus is located in the borough of Westminster (Fig. 1), characterised by heavy traffic in all directions, and a dense cover of buildings, except to the south where the River Thames is situated^20, 21^. A Picarro G2301 cavity ringdown spectrometer (CRDS) was connected to the inlet on the tower to measure continuous CH_4_ and CO_2_ mole fractions. CH_4_ and CO_2_ mole fractions of the samples collected were measured in the RHUL laboratory using a Picarro G1301 CRDS, calibrated against NOAA (National Oceanic and Atmospheric Administration) WMO-2004A and WMO-X2007 reference scales respectively. Comparison between the continuous measurements performed by the Picarro G2301 at KCL and the laboratory measurements of the sampled bags confirmed the consistency between the mole fractions measured by the two instruments.

Diurnal studies were conducted in both summer and winter (when the Picarro instrument was available) to identify the isotopic variability of methane emissions. CH_4_ and CO_2_ continuous trends measured in central London were compared with those measured in Egham, located 32 km WSW from the centre of London (Fig. 1), and receiving air masses from the London region when easterly winds occur. Wind speed and direction averaged every 30 minutes were measured with a weather station (Vaisala WXT520) fitted to the air-sampling tower.

### CH_4_ isotopic analysis

δ^13^C isotopic values of samples collected were measured by CF GC-IRMS (Continuous Flow Gas Chromatography Isotope Ratio Mass Spectrometry) in per mil on the V-PDB scale to high precision (0.05‰)^14^ in the RHUL greenhouse gas laboratory. The source isotopic signature was calculated by Keeling plot analysis, which given a constant background, assumes that the source isotopic value is defined by the intercept of the regression line that correlates the isotopic measurements (y values) with the associated inverse of mole fractions (x values)^22^. The error on the intercept was calculated by the BCES (Bivariate Correlated Errors and intrinsic Scatter) estimator^23^, a statistical procedure that accommodates errors in both variables and accounts for the variability of the error magnitude through the measurements. The intercept of the Keeling plot based on samples collected during the methane build-up observed in diurnal studies represents the δ^13^C signature of emissions from a methane source or mixture of sources responsible for the high mole fraction levels.

To calculate one source isotopic signature from all diurnal studies a Miller-Tans plot^24^ was used. This allows for variable background mole fraction using the following:

$$\delta {C}_{a}-{\delta }_{bg}{C}_{bg}={\delta }_{s}({C}_{a}-{C}_{bg})$$

where *a* represents the atmosphere, *bg* the background and *s* the source. C and δ are the mole fraction and isotopic ratio, respectively. The data are plotted so δ_s_ is the slope of the regression line representing the flux-weighted average of sources.

### Mobile Measurements

Multiple surveys using the Picarro mobile system (described by Zazzeri *et al*.^16^) in central London and the London borough of Hounslow were conducted to detect methane plumes at near ground level, evaluate the spatial allocation of sources suggested by the inventories and verify the occurrence of those methane sources identified through diurnal studies in central London. The system has a mobile Picarro G2301 CRDS in a vehicle for continuous CH_4_ and CO_2_ measurements, with an air inlet and a GPS receiver on the vehicle roof (1.7 m). Mole fractions are displayed in real time, allowing identification of methane plumes during the survey. Air samples for isotopic analysis are collected whenever a broad and consistent plume is detected, by stopping the vehicle and using a diaphragm pump connected to a second air inlet to fill a 3 L Tedlar bag.

## Results

Comparison between the weighted δ^13^C isotopic value calculated with emission inventories, using the typical isotopic signatures assigned to methane source categories, and the isotopic signal measured in central London by Keeling plot analysis, gives insight into the relative importance of methane sources in that area and on the accuracy of the source distribution provided by inventories. Diurnal studies in central London allowed measurement of the mean atmospheric isotopic signal, and analysis of the wind pattern permits identification of the major emission wind sectors.

### The London isotopic source mix: diurnal studies

**Figure 2:**
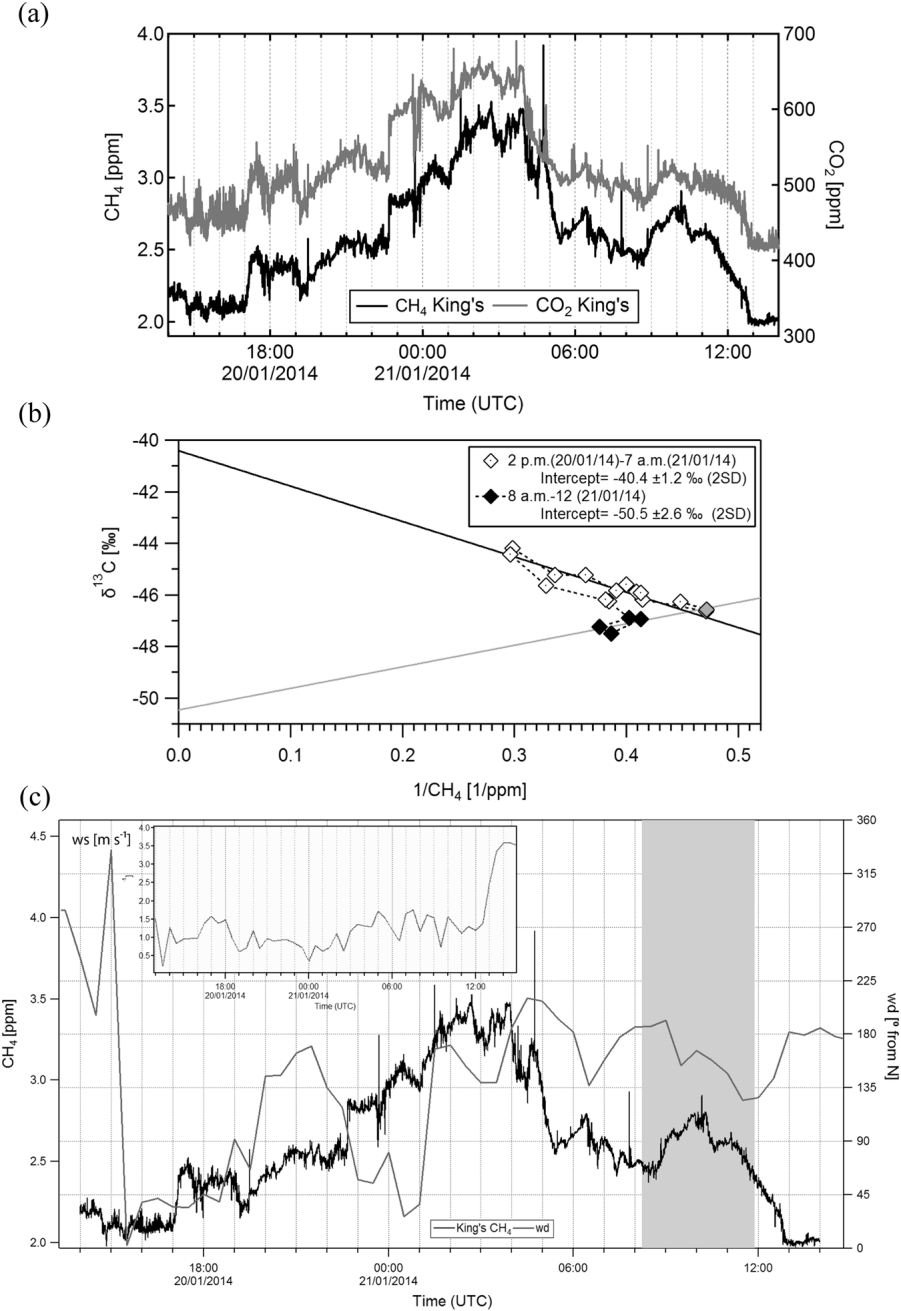
Observations at KCL roof on the 20^th^ and 21^st^ January 2014: (**a**) CH_4_ and CO_2_ mole fractions profiles (**b**) Keeling plots based on the samples collected during the 20^th^ and the early morning of the 21^st^ January 2014 (white markers), during the morning of the 21^st^ January 2014 (black markers), and background (grey marker) with the order of sampling (dotted line). (**c**) Wind direction (grey line), wind speed (line, inset graph) and CH_4_ mole fractions (black line). Time interval of those samples that are thought to be associated with a biogenic CH_4_ source indicated by grey shading.

Continuous CH_4_ and CO_2_ mole fractions were measured from the roof of KCL, in central London (Fig. 1), from 10 December 2013 to 31 January 2014 and from 24 July to 11 August 2014. Herein the diurnal study of 20^th^ to 21^st^ January 2014 is presented, when methane emissions were trapped overnight in the lower boundary layer under typical winter high-pressure conditions. Mole fractions peaked at 3.5 ppm during the night, with a second peak of 2.8 ppm at 10 a.m. on the 21^st^ January (Fig. 2a). After 12 p.m. the wind speed increased and the boundary layer grew, dissipating emissions and leading to the drop in CH_4_ mole fractions close to background value of 2 ppm (Fig. 2c).

While the strong correlation between CO_2_ and CH_4_ (Fig. 2a) gives evidence that boundary layer dynamics are driving the daily trend in mole fractions, the isotopic analysis of samples collected allowed distinction of the methane sources contributing to the CH_4_ build-up.

A ^13^C enriched signature of −40.4 ±1.2‰ for air sampled between 2 p.m. and 7 a.m. suggests a mainly fossil CH_4_ contribution to CH_4_ emissions (Figure 2b). Conversely, the Keeling plot based on the 4 samples collected during the morning build-up on the 21^st^ and the background value (i.e. the lowest mole fraction recorded during the diurnal study), gives a source signature of −50.5 ±2.6‰. According to the wind direction, the methane plume detected in the morning (from 9 a.m. to 11 a.m.) arrived from SSE of KCL (see Fig. 2c).

The measurement of a ^13^C-enriched isotopic signal during the nocturnal build-up and the detection of a biogenic source in the morning before dissipation of the overnight methane build-up following air mixing, was a recurrent pattern (Table 2). The natural gas usage is not constant throughout the day, but peaks in in the early morning (6 to 8 a.m.) and evening (5 to 9 p.m.), when building heating and hot water demands are highest^25^. Emissions derived from gas usage and traffic during evening rush hours are trapped under the nocturnal inversion, leading to an overall ^13^C-enriched signature based on samples collected overnight. The morning biogenic source might be related either to methane emissions from the river, or a very local source (e.g. toilet/sewage vent on the roof) that emits in the morning.

**Table 2:** δ^13^C source signatures calculated with the Keeling plot analysis for each diurnal study.

Date	δ^13^C isotopic signature (‰)	Time of samples included in the Keeling Plot	Number of samples included in the Keeling plot analysis
9^th^–10^th^ Jul 2012	−38.1 ± 4.7	2 p.m. (09/07/2012)–9 a.m. (10/07/2012)	20
2^nd^–3^rd^ Dec 2013	−38.8 ± 1.7	3 p.m. (2/12/2013)–10 a.m. (3/12/2013)	20
10^th^–11^th^ Dec 2013	−36.8 ± 2.2	3 p.m.–10 p.m. (10/12/2013)	5
	−55.2 ± 2.6	2 a.m.–11 a.m. (11/12/2013)	10
20^th^–21^st^ Jan 2014	−40.1 ± 1.2	2 p.m. (20/01/2014)–7 a.m. (21/01/2014)	15
	−50.5 ± 2.6	8 a.m.–12 (21/01/2014)	5
30^th^–31^st^ Jan 2014	−35.9 ± 0.5	5 p.m.–9 p.m. (30/01/2014)	6
	−40.7 ± 0.3	11 p.m. (30/01/2014)–8 a.m. (31/01/2014)	6
	−51.1 ± 0.9	10 a.m.–12 (21/01/2014)	3
12^th^–13^th^–14th March 2014	−42.8 ± 1.0	2 p.m. (12/03/2014)–12 (13/03/2014)	9
	−42.6 ± 0.4	2 p.m. (13/03/2014) – 1 p.m. (14/03/2014)	13
	−50.3 ± 0.7	11:30 p.m. (12/03/2014) – 2 a.m. (13/03/2014)	4
	−53.6 ± 0.5	11:30 p.m. (13/03/2014) – 1 a.m. (14/03/2014)	3
24^th^–25^th^ Jul 2014	−44.2 ± 2.0	2 p.m. (24/07/2014) – 8 a.m. (25/07/2014)	14
	−50.1 ± 2.0	9:30 a.m.–10:30 a.m. (25/07/2014)	3
7^th^–8^th^ Aug 2014	−39.7 ± 1.3	1 p.m. – 9 p.m. (07/08/2014)	9
	−51.6 ± 2.5	5 a.m. – 8 a.m. (08/08/2014)	4

Time is in UTC. Errors are indicated as 2 standard deviations.

**Figure 3:**
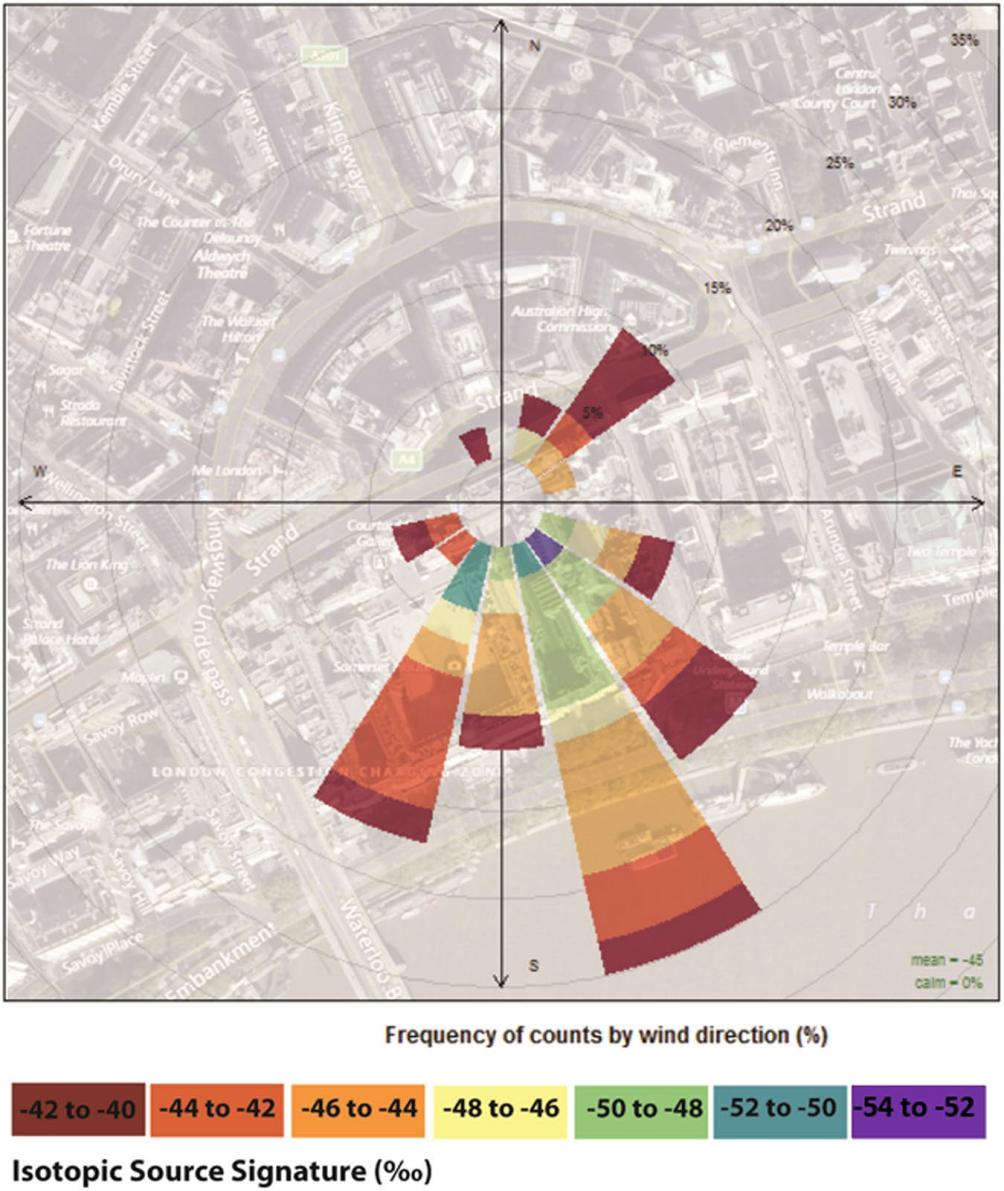
δ^13^C source signature rose for the KCL site for all diurnal campaigns between 2^nd^ December 2013 and 8^th^ August 2014, plotted using OpenAir software. The plot includes all episodes with maximum methane mole fractions at least 100 ppb higher than the background, associated with recorded wind directions (wind speed >0 m s^−1^). Map data: Google, DigitalGlobe.

Figure 3 highlights how the SE wind sector regularly coincides with biogenic methane emissions. All calculated δ^13^C-CH_4_ source signatures for samples collected during these events are divided by wind direction. In this analysis, the source signature is the intercept of a 2-point Keeling plot calculated for samples with and without the source increment. This analysis will not give an accurate calculation of the source signature for the inferred biogenic source, but it indicates the provenance direction of the most ^13^C-depleted methane plumes. Conversely the NE sector shows the largest proportion of ^13^C-enriched source signatures.

**Figure 4:**
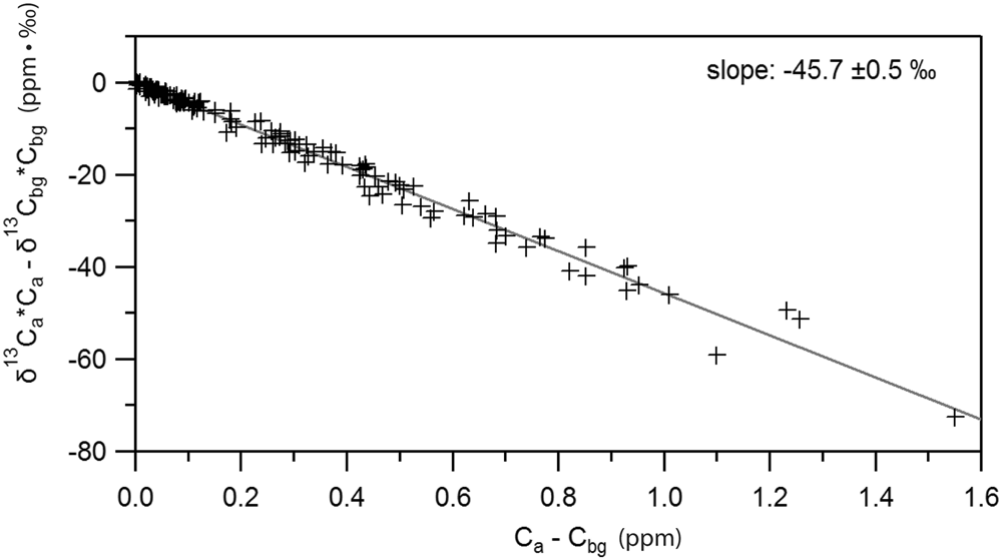
Miller-Tans plot based on all the isotopic values measured and the background value for each diurnal.

The source signature calculated using a Miller-Tans plot based on all the isotopic values measured and the background value for each diurnal period (Fig. 4) is −45.7 ±0.5‰. This value, being isotopically heavier than the background (~−47‰), confirms the primacy of fossil methane emissions in the overall methane budget in central London, an outcome shared by several studies carried out in other big conurbations^26–28^.

Due to the arrangement of buildings surrounding KCL, some very local emissions may interfere with the measurement of surface sources^29^ and occasional CH_4_ mole fraction peaks may occur. Björkegren *et al*.^21^ identified rooftop mechanical ventilation vents and gas heating venting. However, single ^13^C-enriched emission episodes characteristic of combustion (δ^13^C-CH_4_ > −36‰) have not been detected. Furthermore, the Keeling plot intercepts in Table 2 are calculated by considering the overall nocturnal build-up and thus are representative of the mixture of emissions accumulated overnight.

**Figure 5:**
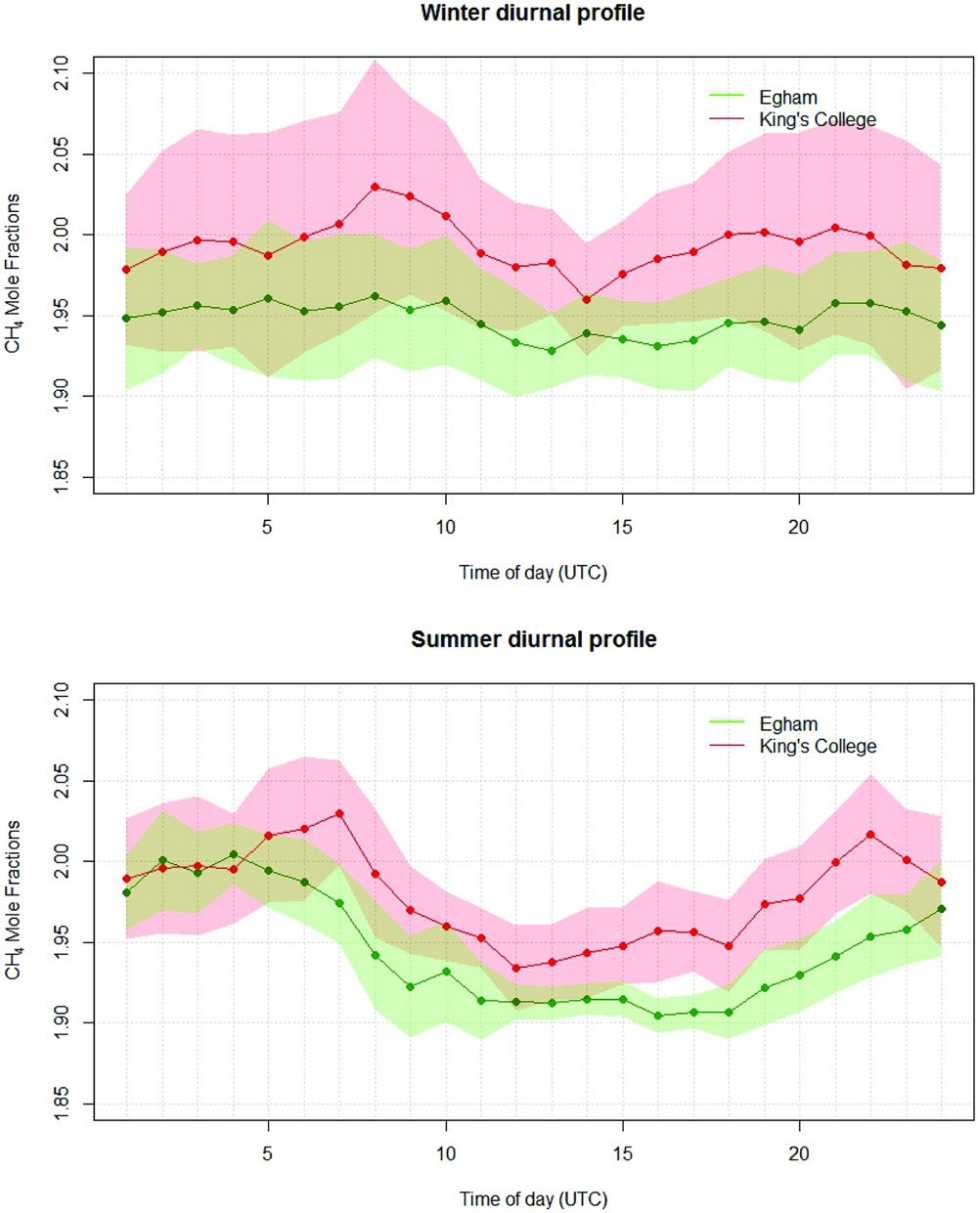
Median hourly diurnal cycle mole fractions in winter (2^nd^ December 2013 to 31^st^ Jan 2014) and summer (24^th^ July 2014 to 6^th^ August 2014) at KCL and Egham. Shading indicates the interquartile range.

Differences in methane emission trends between an urban and peri-urban area can be interpreted using CH_4_ diurnal profiles that use the median values of mole fractions for each hour of the day (Fig. 5) to minimise the influence of residual local CH_4_ plumes, as Rigby *et al*.^30^ did with CO_2_ records for Egham and central London. Continuous mole fraction measurements from KCL and Egham during the diurnal study periods were included in the calculation. Median values of mole fractions recorded at KCL are consistently higher and show a stronger over-night build-up. The median mole fraction at 8 a.m. in London during winter represents the morning build-up that was detected during most of the diurnal studies. During winter the morning reduction in mole fractions occurs later than in summer by 2 hours, as the shorter daylength (reducing surface heating) delays the growth of the boundary layer. Summer diurnal cycles, for the two weeks the Picarro instrument was installed in central London, are more pronounced, reflecting a stronger intensity of the atmospheric turbulence due to a higher surface warming^20, 29^. The difference in CH_4_ mole fractions between the two measurement stations is narrowed, and the diurnal cycles exhibit similar behaviour. Furthermore, the CH_4_ mole fractions characterising the nocturnal build-up in Egham (0–4 a.m.) approach the values recorded in London and are higher than in winter, reflecting enhanced methane emissions perhaps from biological activity such as landfill sites in the region of the Egham station.

### The River Thames: a methane source?

**Figure 6:**
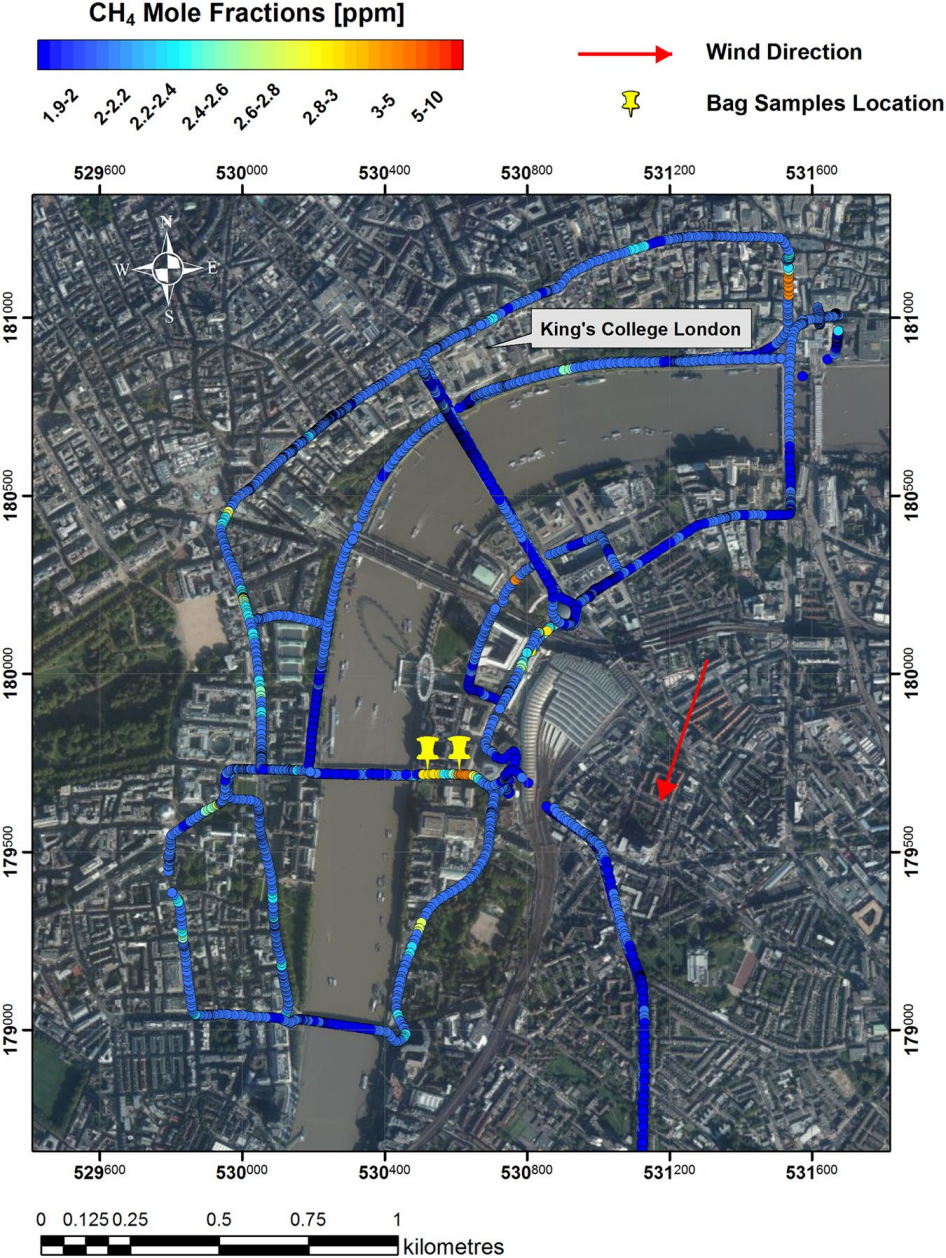
Methane mole fractions (ppm) measured in central London on 20^th^ March 2015 between 8 and 9 a.m. The map was generated using ArcMap 10.2. Base map source: Esri, DigitalGlobe, GeoEye, i-cubed, Earthstar Geographics, CNES/Airbus DS, USDA, USGS, AEX, Getmapping, Aerogrid, IGN, IGP, swisstopo, and the GIS User Community. British National Coordinate System shown.

The consistency of the morning biogenic signature suggested potential emissions from a sewer vent located in proximity to the inlet. A survey with the Picarro mobile system^16^ conducted at low tide in central London on 20^th^ March 2015 between 08:00 and 09:00 UTC (Fig. 6) allowed variations of methane mole fractions along the river banks to be assessed. High methane mole fractions were observed on the eastern ramp of Westminster Bridge, but the Keeling plot intercept of −36.5 ±2.3‰ calculated with the two collected samples and the background, reveals the occurrence of a natural gas leak.

During the survey in March 2015 no other methane plume was intercepted. The very dry conditions characterising that day and the whole previous week might explain the absence of a biogenic signal.

### Evaluation of local inventories: Camden Town and the London Borough of Hounslow

**Figure 7:**
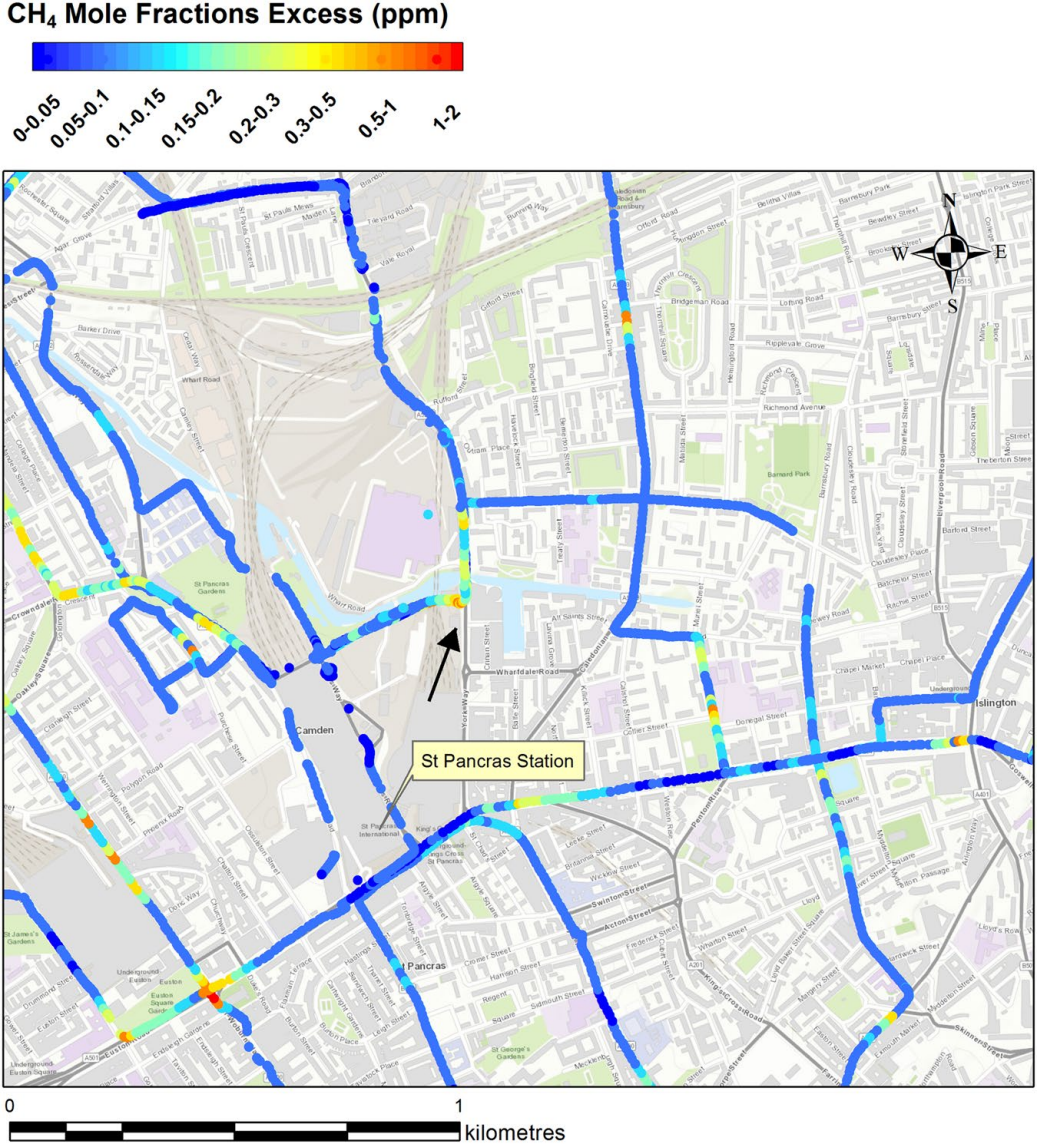
CH_4_ mole fraction excess over the background measured overnight on 14^th^ and 27^th^ April with the Picarro mobile system in the Borough of Camden. The black arrow indicates location of the sample collected. The map was generated using ArcMap 10.2. Base map source: Esri, HERE, DeLorme, Itermap, increment Corp., GEBCO, USGS, FAO, NPS, NRCAN, GeoBase, IGN, Kadaster NL, Ordinance Survey, Esri Japan, METI, Esri China (Hong Kong), swisstopo, MapmyIndia, OpenstreetMap contributors, and the GIS User Community.

A focus on a restricted area puts emphasis on local emissions and their relative isotopic characterisation. The 4 km^2^ in the emission map (Fig. 1, north of KCL) with high inventory methane emissions from the waste sector, are in the London Borough of Camden. The area was surveyed using the Picarro mobile system on the nights of 14^th^ and 27^th^ April 2016. Figure 7 shows the methane mole fraction excess over the background measured during both surveys. Surveys were conducted overnight to measure nocturnal methane build-up and avoid daytime traffic. Unfortunately, road works and closures restricted access, preventing the vehicle stopping for samples. The one sample collected was near a canal water source (black arrow in Fig. 7). The isotopic analysis revealed a biogenic source signature of −58.3‰. This proves the stagnant canal, with enhanced biological activity and anaerobic conditions, is one of the methane sources in central London.

Of the methane emissions in the inventory for the surveyed area, 95% are categorised as “waste” and 0.8% as “nature” emissions^17^. Methane emissions from the canal could be considered in both categories, but a detailed list of methane sources is not provided in the inventories, and the 95% of emissions from the waste sector remains unjustified given the absence of sewage works and landfill sites in that area, and the absence of a wide methane plume typical of a larger source.

Methane spikes over very short time-intervals (10 s) were recorded mainly next to intersections, where main gas pipes are buried and natural gas leaks are most likely to occur.

The spatial allocation of sources given by 2013 emission inventories for the Borough of Hounslow (Fig. 1) was tested through multiple daytime (10:00 to 16:00 local summer time to ensure inversion height does not influence emissions distribution) surveys with the Picarro mobile system in 2014 and 2015. Subsequent isotopic analysis of samples collected downwind of observed methane plumes revealed the methane provenance.

**Figure 8:**
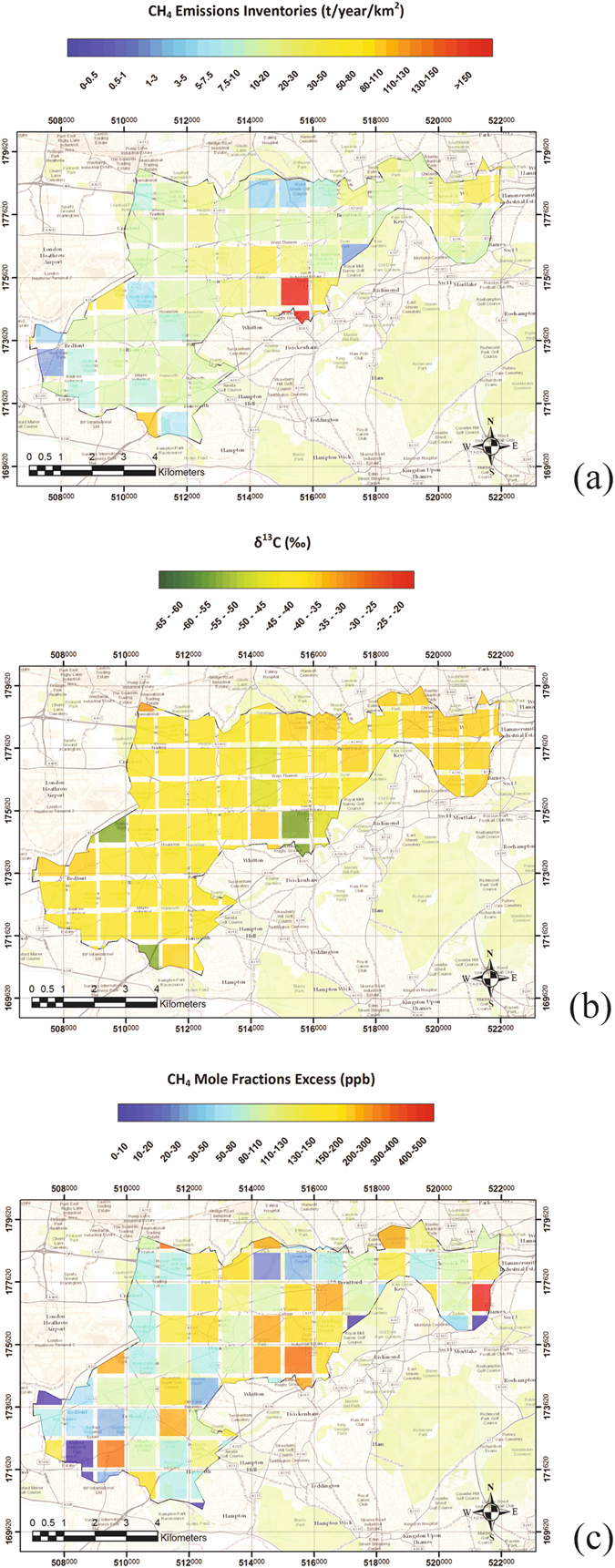
Map of the London borough of Hounslow (**a**) 2013 NAEI emission inventories (**b**) isotopic values calculated from 2013 emission inventories (**c**) values of mole fraction excess relative to the methane baseline in ppb, using measurements from four mobile surveys. The map was generated using ArcMap 10.2. Base map source: Sources: Esri, HERE, DeLorme, Intermap, increment P Corp., GEBCO, USGS, FAO, NPS, NRCAN, GeoBase, IGN, Kadaster NL, Ordnance Survey, Esri Japan, METI, Esri China (Hon Kong), swisstopo, MapmyIndia, OpenStreetMap contributors, and the GIS User Community.

The 2013 inventory emission map for Hounslow (Fig. 8a) has the Mogden sewage works evident (red square). The methane emissions for this have been isotopically characterised by Zazzeri^19^ to have an average value of −53 ±3‰. The isotopic map, created using the same method as Fig. 1, shows the correlation between methane emission hotspots and ^13^C depleted areas, as methane is expected to emit from solid waste disposal and water treatment works. The half green square at south of the borough corresponds to Kempton Park water treatment works (Fig. 8b), whereas the one on the west side includes a cemetery, gravel pit and city farm, considered in the inventories as emitting areas within the waste sector.

The four surveys, carried out in 2014 from 10 a.m. to 4 p.m., during well mixed atmosphere conditions and a consistent wind direction and speed, allowed coverage of the entire borough with a high spatial resolution. Differences between methane mole fractions measured and the background value (mean of the lowest 0.01% of CH_4_ mole fractions) recorded each day of survey were averaged for each km^2^ (Fig. 8c).

The emission map based on inventories and the one based on excess over background allow comparison of source locations. High mole fraction levels were detected next to Mogden sewage works (coordinate 175000 and 515000 in Fig. 8c), confirming this as significant methane source. However, most methane emissions across the borough were in the form of narrow spikes on main roads, suggesting close proximity to gas leaks, with 11 peaks exceeding the background mole fractions by more than 2.5 ppm detected over 260 km (155 miles) of mostly residential roads, a lower ratio per mile than that observed by Phillips *et al*.^15^ in Boston, U.S.A. (3356 leaks in 785 miles).

The hotspot on the east side of the borough (Fig. 8c) is caused by a single gas leak that has been detected during the surveys and does not correspond to a high emission area in the inventory emissions map. Indeed, the localisation of gas leaks is not closely linked to population distribution as in the inventories. In reality their location is difficult to predict, making their emission estimates highly uncertain.

## Discussion

Revised δ^13^C signatures of methane sources in the London region were used to evaluate the source apportionment and spatial allocation made by inventories. Diurnal measurements of methane mole fraction and ^13^C isotopic values on the roof of KCL enabled assessment of the isotopic signal of the source mix in central London, particularly during overnight methane build-up episodes.

The winter source signature recorded during the afternoon and the nocturnal build-up ranges from −40.7 ±0.3‰ to −36.8 ±2.2‰, whereas during summer it spans values between −44.2 ±2.0‰ and −39.7 ±1.4‰. The enhanced photoreaction with hydroxyl radicals in summer, which represents the main sink, and exerts a fractionation effect on methane of −3.9 ±0.4‰^31^, would favour a ^13^C-enrichment of atmospheric methane. The more ^13^C-depleted values recorded in summer suggest instead that higher temperatures might trigger the CH_4_ production from urban biogenic sources (e.g. waste water) and/or the usage of fossil methane is reduced. Indeed, the domestic energy consumption (boilers) in central London varies with time of the year^21, 32, 33^, reaching maximum levels over the end of the year, and it could partially explain the annual shift in the isotopic signal. For instance, new heaters emit prior to ignition a pulse of methane that significantly contributes to the total methane budget when the heating demand is boosted, and gas leaks may increase due to the overpressure on the natural gas distribution system. Fuller *et al*.^34^ showed evidence of wood burning in London as secondary heating during winter, which also would lead to ^13^C-enriched emissions of uncombusted methane. The seasonality of methane emissions caused by variation of the source strength is supported by the study of methane fluxes in central London by Helfter *et al*.^7^.

Diurnal studies at the RHUL Earth Science Department in Egham were performed in the studies of Lowry *et al*.^13^ and Fisher^18^. An isotopic signature of −49.0 to −48.4‰ was recorded for the overall Greater London source mix (area within the orbital motorway), calculated to represent ~20% of methane emissions originating from gas leaks and the rest mainly from waste processing during the 1996–98 period, with δ^13^C-CH_4_ calculated as −52‰ for landfills and −34‰ natural gas emissions. This had changed to a mean δ^13^C source signature of −50.1 ±0.7‰ in 2004 for the London sector (NE to ESE)^18^, again indicating a large contribution of landfill emissions but with a more ^13^C-depleted signature as in-cell gas extraction became the norm – the methane produced in the landfill site and escaping from the surface does not undergo the ^13^C-enrichment associated with partial oxidation through the top-soil. While the overall regional source mix from the London basin was measured during these campaigns in Egham (including many now closed landfill sites in the west London periphery), the main contribution to methane emissions detected at the KCL station might be very local, limited to the London borough of Westminster. Kotthaus and Grimmond^35^ calculated a footprint of ~300–1000 m radius for the KCL tower, using an analytical footprint model based on eddy covariance measurements of fluxes at 13 m above the roof top (a higher level than the inlet used for our measurements). Detection of local fossil fuel plumes in central London might explain measurement of ^13^C-enriched values for the source mix, as biogenic methane emissions are probably sourced further afield in the London region (see Fig. 1).

Comparison between measured isotopic signal (−45.7 ±0.5‰) and the weighted isotopic value calculated for a footprint area of 1 km^2^ using emission inventories and updated δ^13^C signatures (−48‰), demonstrates how the source apportionment suggested by the inventories (53% of emissions from the waste sector and 29% of natural gas) is inconsistent with the predominance of fossil methane resulting from our measurements. Similarly, in Los Angeles, leakages of fossil fuel (e.g. natural gas pipelines, oil refineries and power plants) are identified as major source of methane, while emissions from landfill sites and wastewater treatments do not largely affect methane mole fractions^28^. Levin *et al*.^12^, by comparing the source apportionment of emission inventories with the source partition derived from a 6-year δ^13^C-CH_4_ record, came to the conclusion that CORINAIR90 inventories overestimated emissions from waste management, supported by the fact that the emission factors used for the bottom-up calculation of landfill emissions in Germany were the highest out of all European countries. Therefore, it is commonly stated that inventories might overestimate emissions from the waste sector.

In central London potential biogenic methane emissions cannot be disregarded, but they are most likely coming from the river and canals. A signal from a putative biogenic source has been identified in the KCL record from the SSE direction of the River Thames, related either to a toilet/sewage vent on the roof or the river itself (e.g. a storm relief drain), given that some diurnal studies in winter were carried out after heavy rain periods. Sewage plants in London, which still partly rely on the old collecting system, allow excess flows during heavy rain episodes to be discharged directly into water courses, creating suitable conditions for the anaerobic production of CH_4_^36^. Even though methane sources in river waters are not well established, the presence of elevated dissolved methane in rivers has been proven^37, 38^, with concentrations one or two orders higher than open ocean. In particular, Middelburg *et al*.^39^, in a study of methane distribution in tidal estuaries in Europe, observed that Thames estuary has one of the highest riverine methane concentrations. However, implications for the atmospheric methane budget are not clear, given that air-water exchange coefficients in rivers and the methane flow into the atmosphere are calculated following disparate approaches^40, 41^. Emissions from the Thames need further investigation, and a continuous high-precision isotopic record in central London would definitely provide insight into the interpretation of the urban source mix.

Mobile surveys did not always confirm the methane source emission distribution suggested by the inventories. Isotopic maps developed by converting 1 km^2^ emission estimates to isotopic values, show whether emissions hotspots in the inventories are related to biogenic methane (i.e. from agricultural and waste sector) or fossil fuel sources (i.e. gas leaks and combustion sources). While the expected methane emissions from sewage works were identified both by inventory location and isotopes, most methane peaks were associated with gas leaks that are incorrectly spatially disaggregated, and possibly underestimated in the inventories.

By surveying Camden, one of the suggested methane hotspots in central London, methane emissions from the canal were detected, but these are inconsistent with the 95% of waste emissions suggested for that area.

In conclusion, even though only a qualitative evaluation of inventories has been achieved, since a quantitative evaluation of inventories can be obtained only through flux measurements or atmospheric inversion modelling using mole fraction measurements^42^, this study shows how methane emissions are spatially not well understood and relative proportions by source type not well constrained. The NAEI emission maps are used to support a variety of Government policy works at national scale, in particular as input for air pollution modelling studies. The source distribution is developed using a set of statistics for a specific sector (e.g. energy use, industrial production and employment data, housing and population data) and, as demonstrated in this study, can be highly inaccurate. Therefore, high precision stable isotope analysis of atmospheric methane measurements can be used to give a better understanding of urban and peri-urban sources, necessary for the accomplishment of methane reduction targets.

## Electronic supplementary material

Supplementary information.
