# Supplementary material for: Evaluating methane inventories by isotopic analysis in the London region
Source: Sci Rep. 2017 Jul 7;7:4854. doi: 10.1038/s41598-017-04802-6 (PMC5501781; doi:10.1038/s41598-017-04802-6)
Supplement: Supplementary file 1 — Supplementary information. [file 41598_2017_4802_MOESM1_ESM.pdf]

The automatic sampler

Evaluating methane inventories by isotopic analysis in the London region

G. Zazzeri[1*], D. Lowry[2], R.E. Fisher[2], J.L. France[3], M. Lanoisellé[2], C.S.B. Grimmond[4] and E.G. Nisbet[2*]

[1] Royal Holloway University of London, Egham Hill, Egham, Surrey TW20 0EX 6 (now at Imperial College London, Kensington, London SW7 2AZ)

[2] Royal Holloway University of London, Egham Hill, Egham, Surrey TW20 0EX 6

[3] University of East Anglia, Norwich Research Park, Norwich, Norfolk NR4 7TJ

[4] University of Reading, Reading RG6 6UA

*Corresponding authors

The development of an automatic sampler allowed the collection of up to 20 air samples by setting the start time, filling time and interval between samples remotely. Figure 1a shows the automatic sampler after one complete sampling session. The system has been built on two levels (10 0-4-bar pressure electronically actuated solenoid valves on the upper level and 10 on the bottom one) to attach either 20 tanks or bags. The inflow of outside air is controlled by a KNF-Neuberger diaphragm pump (7 l/min of flow rate) on the inlet line, connected to the air inlet through a ¼” O.D. nylon tube. The air line is flushed for a fixed time of 20 seconds before the valve opens for the collection of each sample, and after the pre-set filling time the valve is immediately closed. Tedlar or Flexfoil bags (SKC Ltd., UK) were found suitable for these studies as they can safely preserve the air collected without leaking during the sampling time and until collection from the site (normally within 24 hours of completion), and for ease of sample transport. A filling time of ~30 seconds was set for each 3 L bag. Once the internet connection was established, the whole system could be started remotely and the sampling cycle run with no interruption until after the collection of the 20th sample. The valves, transducer, pump and sampling software are controlled by a Raspberry Pi computer inside a Pelican case.

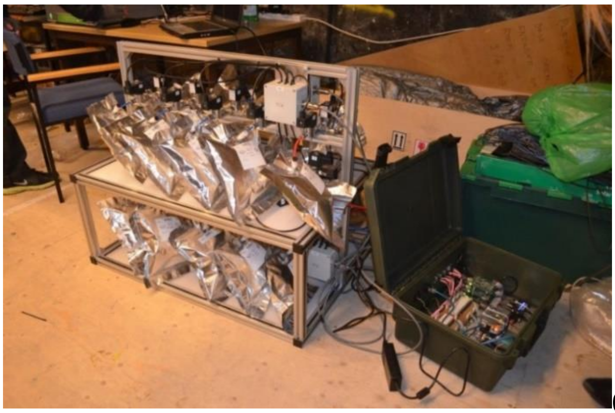

(a)

Manifold 3-D View

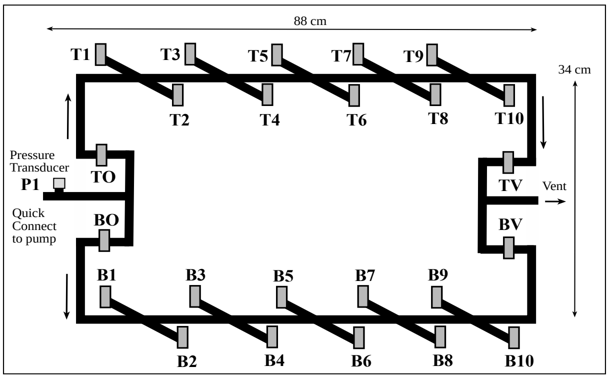

(b)

Figure 1 a) Automatic sampler after one complete sampling session b) scheme of automatic sampler
